# Supplementary material for: Conversion of random X-inactivation to imprinted X-inactivation by maternal PRC2
Source: eLife. 2019 Apr 2;8:e44258. doi: 10.7554/eLife.44258 (PMC6541438; doi:10.7554/eLife.44258)
Supplement: Supplementary file 1. — Statistical comparisons of EED and H3K27me3 IF intensities between all genotypes at each embryonic stage analyzed in Figure 2 (Two-tailed Student’s T-test). [file elife-44258-supp1.docx]

**Pairwise Analysis of IF Intensity Data in Figure 2**

**(Student’s Two-tailed T-test)**

| **~2-cell embryos - EED** | | | | | |
| --- | --- | --- | --- | --- | --- |
|  | *Eed*^fl/fl^ | *Eed*^fl/-^/ *Eed*^-/-^ | *Eed*^m-/-^ | *Eed*^mz-/-^ |  |
| *Eed*^fl/fl^ |  |  |  |  |  |
| *Eed*^fl/-^ / *Eed*^-/-^ | 0.58 |  |  |  |  |
| *Eed*^m-/-^ | 5 x 10^-5^ | .02 |  |  |  |
| *Eed*^mz-/-^ | 2 x 10^-6^ | 3 x 10^-3^ | .88 |  |  |

**~2-cell embryos - H3K27me3**

|  | *Eed*^fl/fl^ | *Eed*^fl/-^/ *Eed*^-/-^ | *Eed*^m-/-^ | *Eed*^mz-/-^ |
| --- | --- | --- | --- | --- |
| *Eed*^fl/fl^ |  |  |  |  |
| *Eed*^fl/-^ / *Eed*^-/-^ | .28 |  |  |  |
| *Eed*^m-/-^ | 4 x 10^-4^ | 1 x 10^-4^ |  |  |
| *Eed*^mz-/-^ | 8 x 10^-6^ | 1 x 10^-5^ | .18 |  |

**~4-cell embryos - EED**

|  | *Eed*^fl/fl^ | *Eed*^fl/-^/ *Eed*^-/-^ | *Eed*^m-/-^ | *Eed*^mz-/-^ |
| --- | --- | --- | --- | --- |
| *Eed*^fl/fl^ |  |  |  |  |
| *Eed*^fl/-^ / *Eed*^-/-^ | 3 x 10^-3^ |  |  |  |
| *Eed*^m-/-^ | 3 x 10^-3^ | .04 |  |  |
| *Eed*^mz-/-^ | 2 x 10^-8^ | 2 x 10^-6^ | 2 x 10^-4^ |  |

**~4-cell embryos - H3K27me3**

|  | *Eed*^fl/fl^ | *Eed*^fl/-^/ *Eed*^-/-^ | *Eed*^m-/-^ | *Eed*^mz-/-^ |
| --- | --- | --- | --- | --- |
| *Eed*^fl/fl^ |  |  |  |  |
| *Eed*^fl/-^ / *Eed*^-/-^ | .08 |  |  |  |
| *Eed*^m-/-^ | 4 x 10^-3^ | .06 |  |  |
| *Eed*^mz-/-^ | 2 x 10^-6^ | 3 x 10^-5^ | .01 |  |

**~8-cell embryos - EED**

|  | *Eed*^fl/fl^ | *Eed*^fl/-^/ *Eed*^-/-^ | *Eed*^m-/-^ | *Eed*^mz-/-^ |
| --- | --- | --- | --- | --- |
| *Eed*^fl/fl^ |  |  |  |  |
| *Eed*^fl/-^ / *Eed*^-/-^ | 2 x 10^-4^ |  |  |  |
| *Eed*^m-/-^ | 5 x 10^-4^ | .67 |  |  |
| *Eed*^mz-/-^ | 8 x 10^-8^ | .02 | .01 |  |

**~8-cell embryos - H3K27me3**

|  | *Eed*^fl/fl^ | *Eed*^fl/-^/ *Eed*^-/-^ | *Eed*^m-/-^ | *Eed*^mz-/-^ |
| --- | --- | --- | --- | --- |
| *Eed*^fl/fl^ |  |  |  |  |
| *Eed*^fl/-^ / *Eed*^-/-^ | .48 |  |  |  |
| *Eed*^m-/-^ | .01 | .076 |  |  |
| *Eed*^mz-/-^ | 5 x 10^-7^ | 1 x 10^-3^ | .03 |  |

**~16-cell embryos - EED**

|  | *Eed*^fl/fl^ | *Eed*^fl/-^ | *Eed*^-/-^ | *Eed*^m-/-^ | *Eed*^mz-/-^ |
| --- | --- | --- | --- | --- | --- |
| *Eed*^fl/fl^ |  |  |  |  |  |
| *Eed*^fl/-^ | .29 |  |  |  |  |
| *Eed*^-/-^ | 6 x 10^-4^ | 3 x 10^-5^ |  |  |  |
| *Eed*^m-/-^ | 7 x 10^-4^ | 5 x 10^-5^ | .44 |  |  |
| *Eed*^mz-/-^ | 5 x 10^-4^ | 4 x 10^-5^ | .01 | .08 |  |

**~16-cell embryos - H3K27me3**

|  | *Eed*^fl/fl^ | *Eed*^fl/-^ | *Eed*^-/-^ | *Eed*^m-/-^ | *Eed*^mz-/-^ |
| --- | --- | --- | --- | --- | --- |
| *Eed*^fl/fl^ |  |  |  |  |  |
| *Eed*^fl/-^ | .14 |  |  |  |  |
| *Eed*^-/-^ | .30 | 9 x 10^-3^ |  |  |  |
| *Eed*^m-/-^ | 5 x 10^-3^ | 2 x 10^-4^ | 6 x 10^-4^ |  |  |
| *Eed*^mz-/-^ | 6 x 10^-3^ | 9 x 10^-4^ | 5 x 10^-4^ | 5 x 10^-3^ |  |
