# Supplementary material for: Conversion of random X-inactivation to imprinted X-inactivation by maternal PRC2
Source: eLife. 2019 Apr 2;8:e44258. doi: 10.7554/eLife.44258 (PMC6541438; doi:10.7554/eLife.44258)
Supplement: Supplementary file 3. — Statistical comparisons of percent allelic expression from the paternal X-chromosome and normalized expression from the paternal- or maternal-X of all genotypes. [file elife-44258-supp3.docx]

| **Pairwise Analysis of RNA-Seq Data**  **% Allelic Expression from Paternal-X – Welch’s Two Tailed T-test** | | | | | |
| --- | --- | --- | --- | --- | --- |
|  | *Eed*^fl/fl^ | *Eed*^fl/-^ | *Eed*^-/-^ | *Eed*^m-/-^ | *Eed*^mz-/-^ |
| *Eed*^fl/fl^ |  |  |  |  |  |
| *Eed*^fl/-^ | 0.42 |  |  |  |  |
| *Eed*^-/-^ | 0.83 | 0.63 |  |  |  |
| *Eed*^m-/-^ | **0.001** | **0.002** | **0.03** |  |  |
| *Eed*^mz-/-^ | **0.04** | **0.005** | **0.01** | **0.02** |  |
|  |  |  |  |  |  |
| **Normalized Allelic Expression from Paternal-X**  **Student’s Two-tailed T-test** | | | | | |
|  | *Eed*^fl/fl^ | *Eed*^fl/-^ | *Eed*^-/-^ | *Eed*^m-/-^ | *Eed*^mz-/-^ |
| *Eed*^fl/fl^ |  |  |  |  |  |
| *Eed*^fl/-^ | 0.42 |  |  |  |  |
| *Eed*^-/-^ | 0.83 | 0.63 |  |  |  |
| *Eed*^m-/-^ | **0.001** | **0.002** | **0.03** |  |  |
| *Eed*^mz-/-^ | **0.04** | **0.005** | **0.02** | **0.02** |  |
|  |  |  |  |  |  |
| **Normalized Allelic Expression from Maternal-X**  **Student’s Two-tailed T-test** | | | | | |
|  | *Eed*^fl/fl^ | *Eed*^fl/-^ | *Eed*^-/-^ | *Eed*^m-/-^ | *Eed*^mz-/-^ |
| *Eed*^fl/fl^ |  |  |  |  |  |
| *Eed*^fl/-^ | 0.74 |  |  |  |  |
| *Eed*^-/-^ | 0.71 | 0.96 |  |  |  |
| *Eed*^m-/-^ | **0.03** | 0.05 | **0.04** |  |  |
| *Eed*^mz-/-^ | **0.002** | 0.09 | 0.08 | 0.53 |  |
|  |  |  |  |  |  |
