# Supplementary material for: Conversion of random X-inactivation to imprinted X-inactivation by maternal PRC2
Source: eLife. 2019 Apr 2;8:e44258. doi: 10.7554/eLife.44258 (PMC6541438; doi:10.7554/eLife.44258)
Supplement: Supplementary file 4. — Statistical comparisons of Pyrosequencing data of Xist, Rnf12, Atrx, and Pgk1 RNAs in embryos of all genotypes. [file elife-44258-supp4.docx]

| **Pairwise Analysis of Pyrosequencing Data in Figures 3 & 4**  **(Welch’s Two-Tailed T-test)** | | | | | |
| --- | --- | --- | --- | --- | --- |
| ***Xist*** |  |  |  |  |  |
|  | *Eed*^fl/fl^ | *Eed*^fl/-^ | *Eed*^-/-^ | *Eed*^m-/-^ | *Eed*^mz-/-^ |
| *Eed*^fl/fl^ |  |  |  |  |  |
| *Eed*^fl/-^ | 0.21 |  |  |  |  |
| *Eed*^-/-^ | 0.16 | 0.17 |  |  |  |
| *Eed*^m-/-^ | **<0.0001** | **<0.0001** | **<0.0001** |  |  |
| *Eed*^mz-/-^ | **<0.0001** | **0.002** | **<0.0001** | 0.32 |  |
|  |  |  |  |  |  |
| ***Rnf12*** |  |  |  |  |  |
|  | *Eed*^fl/fl^ | *Eed*^fl/-^ | *Eed*^-/-^ | *Eed*^m-/-^ | *Eed*^mz-/-^ |
| *Eed*^fl/fl^ |  |  |  |  |  |
| *Eed*^fl/-^ | 0.89 |  |  |  |  |
| *Eed*^-/-^ | 0.10 | 0.14 |  |  |  |
| *Eed*^m-/-^ | **0.0001** | **0.0001** | **0.0001** |  |  |
| *Eed*^mz-/-^ | **0.002** | **0.0002** | **0.002** | 0.35 |  |
|  |  |  |  |  |  |
| ***Atrx*** |  |  |  |  |  |
|  | *Eed*^fl/fl^ | *Eed*^fl/-^ | *Eed*^-/-^ | *Eed*^m-/-^ | *Eed*^mz-/-^ |
| *Eed*^fl/fl^ |  |  |  |  |  |
| *Eed*^fl/-^ | 0.77 |  |  |  |  |
| *Eed*^-/-^ | 0.25 | 0.49 |  |  |  |
| *Eed*^m-/-^ | **0.04** | **0.01** | **0.01** |  |  |
| *Eed*^mz-/-^ | **0.001** | **0.002** | **0.0009** | 0.12 |  |
|  |  |  |  |  |  |
| ***Pgk1*** |  |  |  |  |  |
|  | *Eed*^fl/fl^ | *Eed*^fl/-^ | *Eed*^-/-^ | *Eed*^m-/-^ | *Eed*^mz-/-^ |
| *Eed*^fl/fl^ |  |  |  |  |  |
| *Eed*^fl/-^ | 0.16 |  |  |  |  |
| *Eed*^-/-^ | 0.07 | 0.79 |  |  |  |
| *Eed*^m-/-^ | 0.83 | 0.19 | 0.07 |  |  |
| *Eed*^mz-/-^ | **<0.0001** | **0.0006** | **0.0003** | **<0.0001** |  |
