# Supplementary material for: Conversion of random X-inactivation to imprinted X-inactivation by maternal PRC2
Source: eLife. 2019 Apr 2;8:e44258. doi: 10.7554/eLife.44258 (PMC6541438; doi:10.7554/eLife.44258)
Supplement: Supplementary file 5. — Primer sequences for Pyrosequencing, RT-PCR, genomic PCR, and allele-specific RNA FISH probes; and, antibody information. [file elife-44258-supp5.docx]

| **Pyrosequencing Primers** | | | |
| --- | --- | --- | --- |
| **Gene** | **RT Primer (Forward)** | **RT Primer (Reverse)** | **Pyrosequencing Primer (Sequencing)** |
| *Xist* | CAAGAAGAAGGATTGCCTGGATTT | 5′-biotin-GCGAGGACTTGAAGAGAAGTTCTG | CAAACAATCCCTATGTGA |
| *Atrx* | ATAGCTTCAGATTCTGATGAAACC | 5′-biotin-ACATCGTTGTCACTGCCACTT | TAAGCTCAGATGAAAAGA |
| *Pgk1* | TTTCCGAGCCTCACTGTCC | 5’-biotin-CTTTAGCGCCTCCCAAGA | GTCCAGAGCGACCCT |
| *Rnf12* | 5′-biotin-TGCAGCCAACAAGTGAAATTCC | TATCTGCTGTCTCAGGGTCACATG | TAGAACTTCCTTCAGGC |

| **RT-PCR Primers** | | |
| --- | --- | --- |
| **Gene** | **RT Primer (Forward)** | **RT Primer (Reverse)** |
| *Xist* | GACAACAATGGGAGCTGGTT | CCAGGCAATCCTTCTTCTTG |
| *Eed* | ACCCTCTGGTGTTTGCAACT | GCCTAACCATCGCACACAAT |
| *Pgk1* | GAAGGGAAGGGAAAAGATGC | TGTGCCAATCTCCATGTTGT |
| *Atrx* | GGGATTGCTGCTGTGAGTCT | CCACCATCTTCTTGCCATCT |
| *Rnf12* | GAGCCCCGATGAAAATAGAGC | GGTCGGCACTTCTGTTACTGC |

| **Genomic DNA PCR Primers** | |
| --- | --- |
| **Primer Name** | **Sequence** |
| Eed 5' | GGACTCATCCTCTGGTAGAGCAGC |
| Eed 3' | CCCAAGATCATTACCCCAGA |
| Eed R | TCAATTGGTGGGTTTTGGAT |
| *XY_F* | CCGCTGCCAAATTCTTTGG |
| *XY_R* | TGAAGCTTTTGGCTTTGAG |

| **Allele-specific RNA FISH Probe Coordinates and Sequences** | | | |
| --- | --- | --- | --- |
| **SNP coordinate (mm9)** | **M. musculus specific Probe (with 3' Quasar 570)** | **M. molossinus specific probe (with 3' Quasar 670)** | **Mask Probe** |
| chrX:100664254 | ATCACGCTGAAGACCCAGTTTTCTG | ATCATGCTGAAGACCCAGTTTTCTG | CAGAAAACTGGGTCTT |
| chrX:100669174 | ATGCTGGGAGAACTGCTGTTGTGATG | ATGCCGGGAGAACTGCTGTTGTGATG | CATCACAACAGCAGTT |
| chrX:100676048 | GCTCGGTGGATGAGTTTGAAAGAAAGTAC | GCTCAGTGGATGAGTTTGAAAGAAAGTAC | GTACTTTCTTTCAAACTCA |
| chrX:100676261 | GTGTCGTTGGCATCCAAAATATTCATTG | GTGTTGTTGGCATCCAAAATATTCATTG | CAATGAATATTTTGGATGC |
| chrX:100677431 | CTGCGGCTTCCGCGCAACACC | CTGCTGCTTCCGCGCAACACC | GGTGTTGCGCGG |

| **IF Antibodies** | | | | | |
| --- | --- | --- | --- | --- | --- |
| **Antibody Name** | **Company** | **Catalog Number** | **Dilution** | **Figure** | **Type** |
| Monoclonal EED | ref. Sewalt, R.G. et al. (1998) | | 1:1000 | 1 | primary |
| Monoclonal EED | ref. Sewalt, R.G. et al. (1998) | | 1:2500 | 2 |  |
| Polyclonal H3K27me3 | Millipore | ABE44 | 1:5000 | 1 | primary |
| Polyclonal H3K27me3 | Millipore | ABE44 | 1:25000 | 2 |  |
| Alexa Fluor DαM 555 | Invitrogen | A32773 | 1:300 | 1 | secondary |
| Alexa Fluor DαM 555 | Invitrogen | A32773 | 1:500 | 2 |  |
| Alexa Fluor DαRb 488 | Invitrogen | A21206 | 1:300 | 1 | secondary |
| Alexa Fluor DαRb 488 | Invitrogen | A21206 | 1:500 | 2 |  |
| Alexa Fluor DαRb 647 | Invitrogen | A31573 | 1:300 | 3,4,S2 | secondary |
